# Cucurbitacin E Induces Autophagy-Involved Apoptosis in Intestinal Epithelial Cells

**DOI:** 10.3389/fphys.2020.01020

**Published:** 2020-08-26

**Authors:** Huapei Song, Hehuan Sui, Qiong Zhang, Pei Wang, Fengjun Wang

**Affiliations:** ^1^State Key Laboratory of Trauma, Burn and Combined Injury, Institute of Burn Research, Southwest Hospital, Third Military Medical University (Army Medical University), Chongqing, China; ^2^Department of Pharmacy, Central Hospital of Nanchong, The Second Clinical School of North Sichuan Medical College, Nanchong, China; ^3^Nanchong Key Laboratory of Individualized Drug Therapy, Nanchong, China

**Keywords:** cucurbitacin E, intestinal epithelial cells, apoptosis, autophagy, endoplasmic reticulum stress

## Abstract

Apoptosis plays a crucial role in maintaining the structural and functional integrity of the intestinal epithelial barrier. Autophagy mediates injury to and repair of the intestinal epithelial barrier through multiple pathways in pathophysiological conditions. Our earlier study has found that cucurbitacin E (CuE) regulates the proliferation, migration, and permeability of human intestinal epithelial cells (IECs); however, its effects and mechanisms on apoptosis and autophagy are still unclear. This study reported CuE induced apoptosis and promoted autophagy of IECs in a concentration-dependent manner. The results showed that CuE could inhibit the expression of apoptosis-related protein Bcl-2 and drove activation of caspase-3 and cleavage of its substrate poly (ADP-ribose) polymerase. CuE also facilitated the expression of endoplasmic reticulum stress-related proteins, CHOP and Grp78, and autophagy-related proteins, Beclin1 and LC3, while inhibiting the phosphorylation of AKT and mammalian target of rapamycin (mTOR). An autophagy inhibitor, 3-methyladenine, reduced CuE-induced apoptosis. These results suggest that CuE may induce apoptosis and autophagy in IECs *via* the PI3K/AKT/mTOR signaling pathway and that autophagy following endoplasmic reticulum stress participates in the pro-apoptotic process induced by CuE.

## Introduction

The structural and functional integrity of the intestinal epithelial barrier depends on the presence of healthy epithelial cells and normally functioning paracellular pathways. If the physiological proliferation or apoptosis of human intestinal epithelial cells (IECs) changes, inflammatory cytokines can impair intestinal epithelial barrier function *via* the transepithelial pathway, leading to the occurrence of bacterial translocation, chronic intestinal infection, and even tumors (Peterson and Artis, 2014; Okumura and Takeda, 2017). Autophagy is a cellular self-catabolism process that drives cell survival by degrading and recovering protein aggregates and impaired organelles. It has been shown that autophagy mediates the repair of intestinal epithelial barrier injury *via* multiple pathways, for instance, by regulating intestinal epithelial tight junctions, participating in pathogen clearance, controlling inflammatory signal expression and immune function, and intervening in intestinal mucus secretion and endoplasmic reticulum stress (ERS; Randall-Demllo et al., 2013; Novak and Mollen, 2015; Pott et al., 2018).

In recent years, the medicinal value of cucurbitacin, an effective ingredient in plants of the family Cucurbitaceae, has received increasing attention. Cucurbitacin E (CuE) is a tetracyclic triterpenoid compound extracted from Cucurbitaceae plants. CuE has various pharmacological properties, including anti-inflammatory, antitumor, and antioxidant effects (Attard and Martinoli, 2015). Our recent research results showed that CuE inhibits the proliferation and migration of human IECs by activating cofilin, and in hypoxic conditions, activated cofilin regulates the permeability of the intestinal epithelium by depolymerizing F-actin (Song et al., 2018, 2019). However, the effects of CuE on apoptosis and autophagy in IECs are still unclear and deserve further investigation.

## Materials and Methods

### Materials

Human IEC Caco-2 cells were provided by the Shanghai Institute of Biochemistry and Cell Biology, Chinese Academy of Sciences (Shanghai, China). CuE was purchased from Chenguang Biotech (Baoji, China), with a stock solution prepared in dimethyl sulfoxide and stored at −20°C. Dulbecco’s Modified Eagle’s Medium (DMEM) and fetal bovine serum were from Gibco (USA). Trypsin was from BI. The cell culture incubator was from Thermo (Waltham, MA, USA). The fluorescent dye Hoechst 33258 was from Beyotime (Shanghai, China). TUNEL apoptosis assay kits were from Vazyme. The inverted fluorescence microscope was manufactured by Leica (German). Annexin V-FITC, propidium iodide (PI), and the FACSCalibur flow cytometer were all from BD (USA). The UP-201 ultrasonic tissue/cell disrupter was from TOMY (Japan). Antibodies against caspase-3, poly (ADP-ribose) polymerase (PARP), Bcl-2, CHOP, Grp78, LC3, Beclin1, PI3K, p-PI3K, AKT, p-AKT, mTOR, and p-mTOR were from Cell Signaling (USA). Anti-β-actin antibody and 3-methyladenine (3-MA) were from Sigma (USA). The hypersensitive ECL chemiluminescence kit was from GE (USA). PVDF membrane was manufactured by MILLIPORE (USA). Gel electrophoresis, transfer membrane blocking, and Chemi Doc™ XRS+ imaging systems were all from BIO-RAD (USA).

### Cell Culture

Caco-2 cells were cultured in DMEM containing 100 U/ml penicillin, 100 μg/ml streptomycin, 10% fetal bovine serum, 2 mmol/L glutamine, and 1 mmol/L nonessential amino acids, pH 7.4. The cells were cultured in a saturated humidity incubator with 5% CO_2_ at 37°C. The culture medium was exchanged once every 2–3 days. When 80% confluence was reached, the cells were digested with 0.25% trypsin and 0.53 mmol/L ethylenediaminetetraacetic acid solution and passaged at a ratio of 1:3.

### Annexin V-FITC/PI Flow Cytometry

Caco-2 cells were seeded on a six-well plate and treated with different concentrations of CuE for 24, 48, and 72 h. Then, trypsin was added to harvest the cells. Annexin V-FITC, PI, and 1 × apoptosis binding buffer were mixed at a ratio of 1:1:50. For each sample, 100 μl of Annexin V-FITC/PI was added to label cells for 15 min in the dark at room temperature. Subsequently, each sample was diluted by adding an equal volume of binding buffer and then analyzed on a FACSCalibur flow cytometer. For each sample, 1 × 10^6^ cells were counted, and data were analyzed using ModFit 3.2.

### Western Blot

Western blot analysis was performed in accordance with the methods described in our previous studies (Cao et al., 2013), using β-actin as the loading control. The Caco-2 cells were seeded in a six-well plate and used for experiments after confluence. The cells were treated with 0.001, 0.01, 0.1, 1, and 10 μmol/L CuE for 24 h, with DMEM as the control. Then, the cells were washed once with pre-cold PBS, followed by lysis with SDS-PAGE sample and brief sonication using a sonicator. After centrifuging at 12,000 rpm 4°C for 10 min, the supernatant was collected to boil in water bath for 5 min. Equal amounts of extracted protein from each sample was separated on SDS-PAGE, followed by transferring the proteins to PVDF membrane. The protein blot was blocked with 5% skim milk for 1 h. After incubating each individual protein blot with the corresponding primary antibodies overnight at 4°C, the membranes were washed four times in TBST (15 min each), incubated with the corresponding secondary antibodies at room temperature for 1 h, and washed another four times with TBST. Chemiluminescent reagent was used to develop the protein blots and ChemiDoc XRS system was used for chemiluminescent signal acquisition. Quantity One software (Bio-Rad Laboratories) were used for the result analysis.

### Statistical Analysis

Statistical analyses were performed using SPSS 17.0 Statistics (SPSS Inc., Chicago, IL, USA). All data are expressed as *x̄* ± s. One-way analysis of variance (ANOVA) was used for comparisons among groups. The *t*-test was performed for the comparison of two-sample means. A value of *p* < 0.05 indicated statistical significance.

## Results

### CuE Induced Apoptosis in Caco-2 Cells

**Figure 1:**
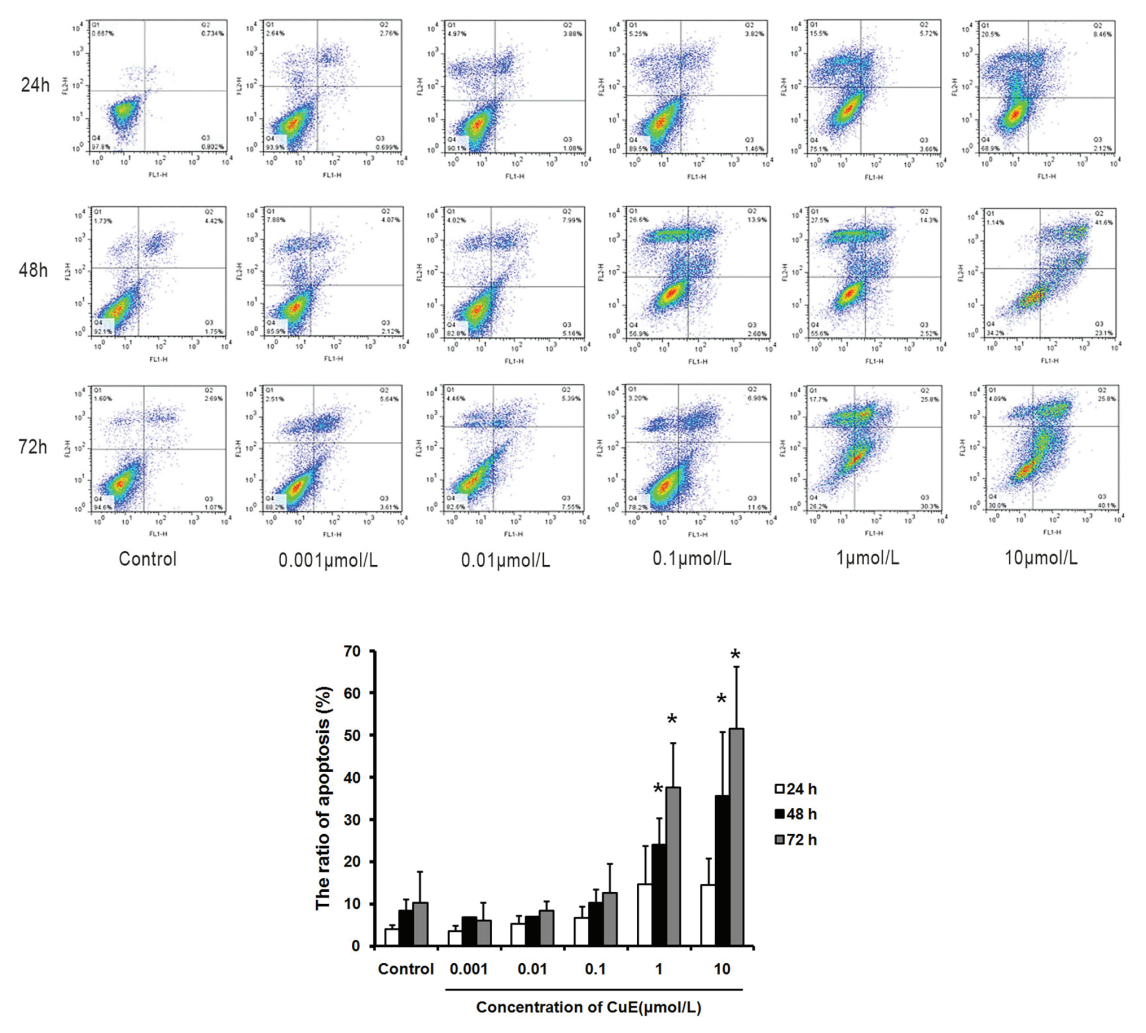
Cucurbitacin E (CuE) induced apoptosis and cell cycle arrest in Caco-2 cells. The cells were treated with 0.001, 0.01, 0.1, 1, and 10 μmol/L CuE for 24, 48, and 72 h, respectively. Cell apoptosis was observed by Annexin V-FITC/PI staining and flow cytometry analysis. ^*^ *p* < 0.05 compared with control group. Data are representative of five similar experiments.

The effect of CuE on Caco-2 cell apoptosis was observed by Annexin V-FITC/PI staining and flow cytometry analysis (Figure 1). Compared with that in the control group, the apoptosis rates gradually increased in the groups treated with 1 μmol/L CuE for 24, 48, and 72 h. Compared with that in the control group, a significant increase in the percentage of apoptotic cells was detected after treatment with 1 μmol/L CuE for 48 h (*p* < 0.05) and 10 μmol/L CuE for 24 h (*p* < 0.05).

### CuE Drove Caspase-3 Activation and PARP Cleavage While Inhibiting Bcl-2 Protein Expression

**Figure 2:**
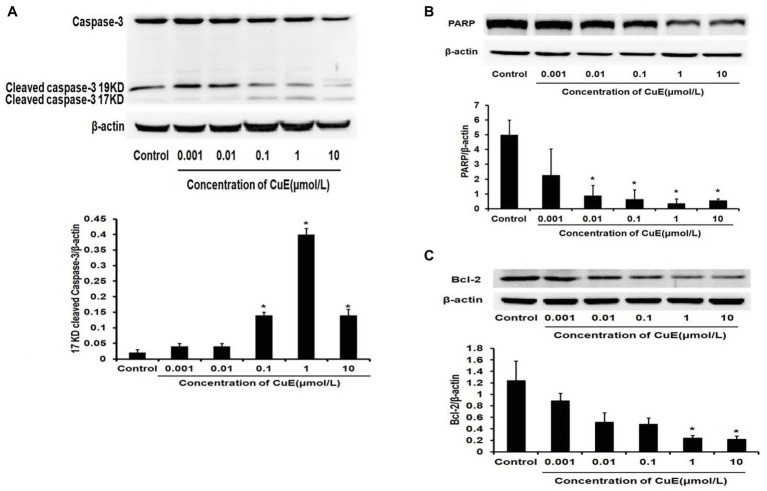
CuE induced changes of apoptosis-related proteins. Expression of caspase-3, cleaved-caspase-3 **(A),** and PARP **(B)** by western blotting, after 24 h of treatment with different concentrations of CuE. **(C)** Expression of Bcl-2 by western blotting, after 24 h of treatment with different concentrations of CuE. β-actin as a loading control. The ratio of gray level of protein bands between target protein and β-actin were showed in *Y*-axis. ^*^ *p* < 0.05 compared with control group. Data are representative of five similar experiments.

The effect of CuE on the expression of the apoptosis-related proteins caspase-3 and PARP in Caco-2 cells was investigated by western blot analyses. After 24 h of treatment with different concentrations of CuE, caspase-3 protein expression decreased to varying degrees, while cleaved-caspase-3 protein expression increased with 0.1, 1, and 10 μmol/L CuE treatments (Figure 2A). Additionally, PARP protein expression in Caco-2 cells decreased to different degrees with 0.01, 0.1, 1, and 10 μmol/L CuE treatments (*p* < 0.05; Figure 2B). These results indicate that CuE induced caspase-3 activation in Caco-2 cells, leading to PARP cleavage. Furthermore, an effect of CuE on Bcl-2 protein expression was observed (Figure 2C). Compared with that in the control group, there was a significant decrease in Bcl-2 protein expression in Caco-2 cells after 24 h of 1 and 10 μmol/L CuE treatment (*p* < 0.05).

### CuE Promoted the Expression of the ERS-Related Proteins CHOP and Grp78

**Figure 3:**
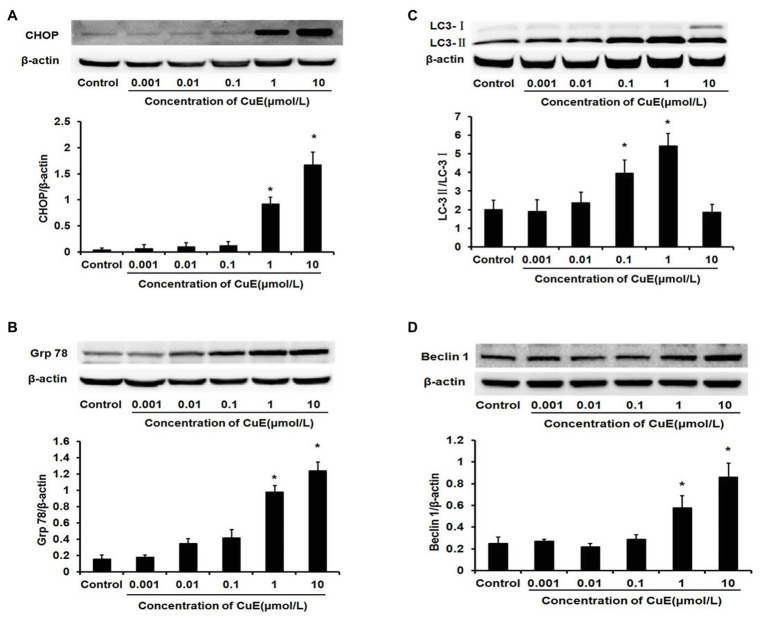
CuE promoted the expression of the endoplasmic reticulum stress (ERS)‐ and the autophagy-related proteins. The Caco-2 cells were treated with 0.001, 0.01, 0.1, 1, and 10 μmol/L CuE for 24 h before western blotting. **(A)** Expression of CHOP with different concentration of CuE. **(B)** Expression of Grp78 with different concentration of CuE. **(C)** Expression of LC3-I and LC3-II with different concentration of CuE. The ratio of LC3-II/LC3-I (*Y* axis) showed the degree of autophagy. **(D)** Expression of Beclin1 with different concentration of CuE. β-actin as a loading control. The ratio of gray level of protein bands between target protein and β-actin were showed in *Y*-axis. ^*^ *p* < 0.05 compared with control group. Data are representative of five similar experiments.

The western blot results for changes in CHOP protein expression in Caco-2 cells after treatment with different concentrations of CuE are illustrated in Figure 3A. CHOP protein expression gradually and slowly increased after administration, and a significant increase in expression was observed in the 1 and 10 μmol/L CuE groups compared with that in the control group (*p* < 0.05). The western blot results for changes in Grp78 protein expression are shown in Figure 3B. Grp78 protein expression exhibited a gradually increasing trend after CuE administration, and the increase in expression was significant in the 1 and 10 μmol/L CuE groups compared with that in the control group (*p* < 0.05).

### CuE Facilitated the Expression of the Autophagy-Related Proteins LC3 and Beclin1

The western blot results for changes in LC3 protein expression in Caco-2 cells after CuE treatment at different concentrations are provided in Figure 3C. Cytosolic LC3-I was enzymatically hydrolyzed into membrane-bound LC3-II. LC3-II/LC3-I ratio has been widely used as an indicator of autophagy (Mizushima and Yoshimori, 2007). Compared with that in the control group, the LC3-II/LC3-I ratio first increased and then decreased in the groups treated with CuE. A significant increase in the ratio was observed in the 0.01, 0.1, and 1 μmol/L CuE groups (*p* < 0.05), with the highest value in the 1 μmol/L CuE group. By contrast, the ratio decreased in the 10 μmol/L CuE group, and the result was even lower than that of the control group. Moreover, compared with that in the control group, Beclin1 protein expression gradually increased in the groups treated with CuE; protein expression in the 1 and 10 μmol/L CuE groups was significantly higher than that in the control group (*p* < 0.05; Figure 3D).

### CuE Inhibited Activation of PI3K/AKT/mTOR in Caco-2 Cells

**Figure 4:**
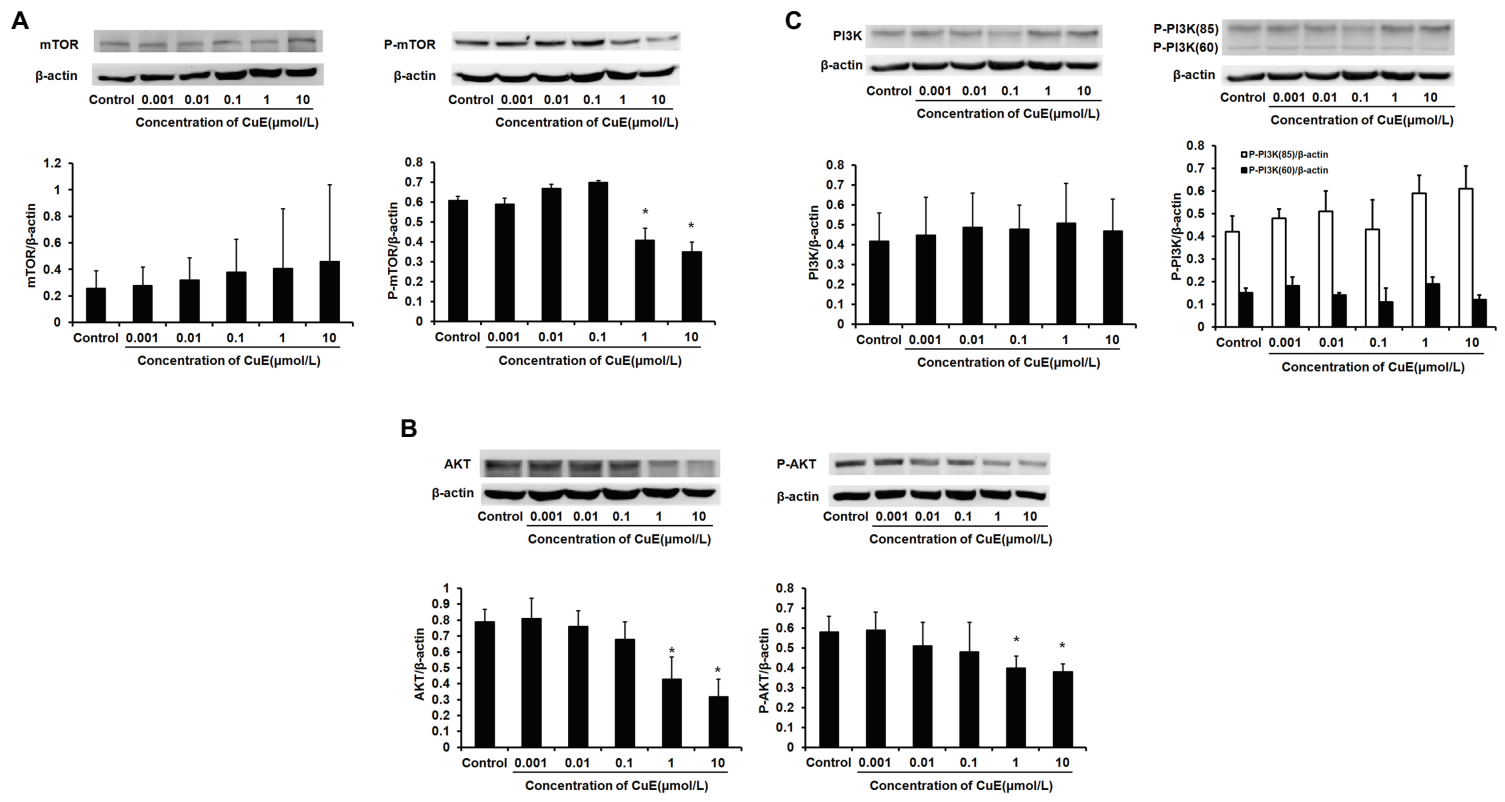
CuE inhibited PI3K/AKT/mTOR pathway. The Caco-2 cells were treated with 0.001, 0.01, 0.1, 1, and 10 μmol/L CuE for 24 h (*X*-axis) before western blotting. **(A)** Expression of mTOR and p-mTOR with different concentration of CuE. **(B)** Expression of AKT and p-AKT with different concentration of CuE. **(C)** Expression of PI3K and p-PI3K with different concentration of CuE. β-actin as a loading control. The ratio of gray level of protein bands between target protein and β-actin were showed in *Y*-axis. ^*^p < 0.05 compared with control group. Data are representative of five similar experiments.

The effect of different concentrations of CuE on mTOR and p-mTOR protein expression in Caco-2 cells after 24 h of treatment was determined by western blot analysis (Figure 4A). It was found that mTOR protein expression did not change as the concentration of CuE increased (*p* > 0.05), but mTOR phosphorylation was inhibited in the 1 and 10 μmol/L CuE groups (*p* < 0.05), relative to that in the control group. Furthermore, the effect of CuE on AKT and p-AKT protein was determined by western blot analysis (Figure 4B). Compared with that in the control group, both AKT and p-AKT protein expressions gradually decreased as the concentration of CuE increased; AKT and p-AKT protein expressions in the 1 and 10 μmol/L groups were significantly lower than that in the control group (*p* < 0.05). Subsequently, the effect of CuE on the expression of an upstream signaling molecule, PI3K, and its phosphorylated protein, p-PI3K, was further investigated (Figure 4C). Compared with that in the control group, total PI3K protein expressions in the groups treated with increasing concentrations of CuE were not significantly different (*p* > 0.05). However, the expression of 85 and 60-kDa p-PI3K proteins changed to varying degrees, albeit not significantly different compared with that in the control group (*p* > 0.05).

### The Autophagy Inhibitor 3-MA Reduced CuE-Induced Apoptosis

**Figure 5:**
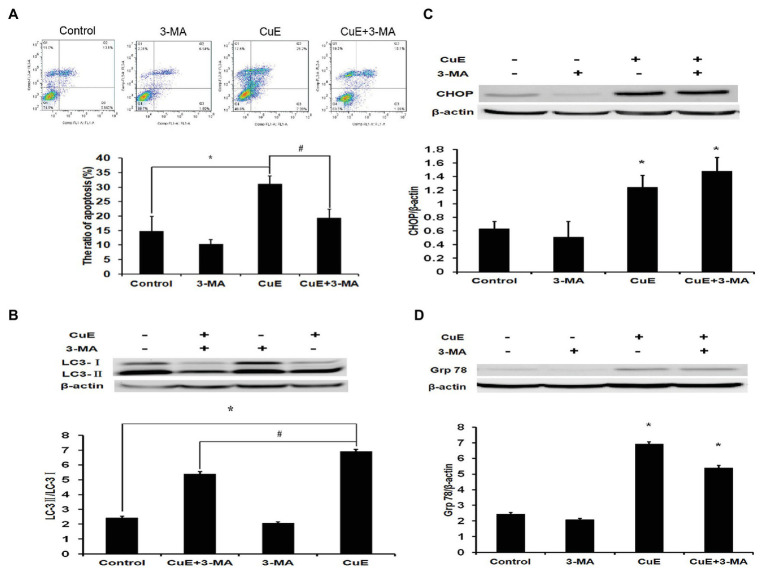
Autophagy involved in CuE-induced cell apoptosis. Caco-2 cells were divided into four groups: control group, 5 mM autophagy inhibitor 3-MA group, 1 μM CuE group, and CuE+3-MA group. **(A)** Flow cytometry assay with Annexin V-FITC/PI staining. **(B)** Expression of LC3 by western blotting. The ratio of LC3-II/LC3-I showed the degree of autophagy. **(C)** Expression of CHOP by western blotting. **(D)** Expression of Grp78 by western blotting. β-actin as a loading control. The ratio of gray level of protein bands between target protein and β-actin were showed in *Y*-axis. ^*^ *p* < 0.05 compared with control group. ^#^ *p* < 0.05 compared with CuE group. Data are representative of five similar experiments.

Based on the experimental results, cells were divided into four groups: control group, autophagy inhibitor 3-MA group (5 mM), CuE group (1 μM), and CuE+3-MA group. The effect of different drugs on Caco-2 cell apoptosis was determined using Annexin V-FITC/PI staining and flow cytometry (Figure 5A). In the CuE group, apoptotic cells significantly increased compared with those in the control group (*p* < 0.05), and in the CuE+3-MA group, apoptotic cells decreased compared with those in the CuE group (*p* < 0.05).

### CuE Combined With 3-MA Increased Hydrolysis of LC3 Protein

After 24 h of treatment with different drug concentrations, changes in LC3 protein expression in Caco-2 cells were determined by western blot analysis (Figure 5B). The LC3-II/LC3-I ratio increased in the CuE group relative to that in the control group, while the LC3-II/LC3-I ratio in the CuE+3-MA group was remarkably lower than that in the CuE group (*p* < 0.05).

### CuE Combined With 3-MA Facilitated CHOP and Grp78 Protein Expression

After 24 h of treatment with different drug concentrations, the changes in CHOP protein expression in Caco-2 were determined by western blot analysis (Figure 5C). Compared with that in the control group, the protein expression did not change significantly in the 3-MA group, while the protein expression significantly increased in the CuE group and the CuE+3-MA group (*p* < 0.05). The western blot results regarding changes in Grp78 protein expression in Caco-2 cells after 24 h of treatment in different groups are provided in Figure 5D. Compared with that in the control group, the protein expression slightly decreased in the 3-MA group (*p* > 0.05) and significantly increased in the CuE group and the CuE+3-MA group (*p* < 0.05).

## Discussion

The structure and function of the intestinal mucosa may be impaired in the case of severe burns and trauma, critical illness, malnutrition, and severe infection or inflammation. This impairment leads to intestinal mucosal barrier dysfunction, intestinal bacterial translocation, and endotoxin invasion, which in turn induces or aggravates local intestinal or systemic inflammatory responses and even causes multiple organ dysfunction in severe cases (Attia et al., 2017; Delorme-Axford and Klionsky, 2018; He et al., 2019). Apoptosis is the process of active cell death and is regulated by free radicals. This process enables the body to clear injured, aged, and useless cells; however, apoptosis itself does not cause injury or inflammation in the microenvironment of the body. Apoptosis is indispensable for maintaining homeostasis in tissues, the dynamic balance of cell populations, and various physiological functions and pathological responses of tissues and organs. It has been shown that apoptosis plays a crucial role in intestinal epithelial barrier function, and disorders of apoptosis regulation may lead to the development of inflammatory bowel disease and intestinal tumors (Watson, 2004; Pedersen et al., 2014). Cucurbitacin has a broad range of pharmacological properties, such as anti-inflammatory, antitumor, and hepato-protective effects. Among the numerous known family members of cucurbitacin, CuE is one of the more important members. Caco-2 cell line is the most widely used in the intestinal research in recent years, which has appropriate similarity with healthy intestinal epithelium in morphology and biochemical properties. Especially, among the human intestinal cell lines, monolayers of Caco-2 cell play the most important and suitable role in the study of permeability of IECs. Thus, it has been chosen as a cell model for our research about the intestinal barrier function. The previous research in Caco-2 cells has found that CuE inhibits the proliferation and migration of IECs *via* activating cofilin, while activated cofilin can increase intestinal permeability. Other studies have shown that cell apoptosis correlates with proliferation and migration (Alenzi, 2004; Erika and Denise, 2004). In the present study, we observed the effect of CuE on apoptosis of Caco-2 cells *in vitro*. The results show that CuE can induce apoptosis in Caco-2 cells and that this effect is related to the concentration and duration of CuE treatment; the higher the concentration is and the longer the duration of treatment is the more evident the apoptosis induction effect of CuE is. Similar to our results, other studies have shown that CuE can induce apoptosis in 95D human lung cancer cells, SAS oral squamous cell carcinoma cells, and SW527 breast cancer cells. Furthermore, our previous study showed that CuE could induce G2/M cell cycle arrest in Caco-2 cells (Song et al., 2018). Other studies demonstrate that prolonging the cell cycle may lead to secondary apoptosis (Liu et al., 2003). It is suggested that cell cycle arrest might induce apoptosis in Caco-2 cells.

The molecular mechanisms underpinning CuE-induced apoptosis are not very clear. It has been suggested that apoptosis induced by CuE may be related to the inhibition of intracellular STAT3 phosphorylation, an elevation in P53 and P21 levels and a reduction in CDK1 and cyclin B levels (Huang et al., 2012); this phenomenon may also be related to an upregulation of cyclin D1, survivin, Bcl-2, and Mcl-1 expression by CuE (Kong et al., 2014). Moreover, it has been found that CuE induces apoptosis in tumor cells *via* a mitochondrial pathway and the caspase-dependent pathway, whereby caspase-3 plays a vital role. Caspase-3 is a core protease that mediates apoptosis (Hung et al., 2013). The caspase-3 cascade activated by upstream signals such as Bcl family proteins leads to the occurrence of apoptosis by cleaving the substrate PARP. To further clarify the molecular mechanisms underpinning CuE-induced apoptosis in IECs, we determined the effect of CuE on Bcl-2, caspase-3, and PARP protein expression. The results indicate that CuE can inhibit Bcl-2 protein expression, while inducing caspase-3 activation and cleavage of the caspase-3 substrate PARP. Therefore, we propose the following possible mechanism of CuE-induced apoptosis in IECs: CuE inhibits the expression of the anti-apoptotic protein Bcl-2, activates caspase-3, and causes PARP cleavage.

Beclin1, an essential molecule for the formation of autophagosomes, mediates the localization of autophagy-related proteins to phagocytic vesicles and interacts with various proteins to regulate autophagosome formation and maturation (Maejima et al., 2016). To further clarify the feature of autophagy in IECs, we determined the effect of different concentrations of CuE on the expression of Beclin1 and the autophagy marker LC3. The results showed that CuE could promote Beclin1 protein expression and LC3 enzymatic hydrolysis, which suggested that CuE might induce autophagy in IECs. Additionally, the LC3-II/LC3-I ratio decreased in the 10 μmol/L CuE group, which might be correlated with cytotoxicity and substantial amount of cell death induced by high concentration of CuE. ERS, which can cause the accumulation of misfolded or unfolded proteins in the cytoplasm, is also an important factor in the induction of autophagy (Wang and Kaufman, 2016). When ERS is too strong or the duration is too long, it can induce a transient upregulation of autophagy and then activate apoptosis pathways (Kroemer et al., 2010). ERS-mediated autophagy has a dual role: anti-apoptosis and pro-apoptosis. Glucose-regulated protein 78 (Grp78) is a target molecule for the unfolded protein response, and it activates subsequent signaling pathways including PERK, IRE1, and ATF6 (Cao and Kaufman, 2014). During ERS, CHOP activation directly or indirectly mediates pro-apoptotic signals *via* multiple pathways, for instance, by inhibiting the transcription of Bcl-2, upregulating the expression of oxidase 1α in the endoplasmic reticulum, and activating the transcriptional activity of TRB3 (Cheng et al., 2015; Jeon et al., 2018). The results revealed that CuE promoted CHOP and Grp78 protein expression, which demonstrated that ERS was probably involved in CuE-induced autophagy and apoptosis. Studies have indicated that the relationship between PI3K/AKT/mTOR signaling pathway and apoptosis is complex, which regulates apoptosis differently in various diseases and cells (Chaudhuri et al., 2014; Liu et al., 2014). The mTOR pathway, which acts as a sensor of cell nutritional status and stress and growth factor signals, plays a critical role in the regulation of autophagy (Jung et al., 2010). Furthermore, mTOR inhibits autophagy by controlling ULK1 ubiquitylation, self-association, and function through AMBRA1 and TRAF6 (Francesca et al., 2013). In the present study, we further examined the effect of CuE on the PI3K/AKT/mTOR signaling pathway in Caco-2 cells. The results showed that CuE could inhibit AKT and mTOR phosphorylation, which meant that the PI3K/AKT/mTOR signaling pathway might mediate the role of CuE in promoting IEC apoptosis and autophagy.

Increasingly, more studies have evidenced that autophagy and apoptosis are not independent; instead, they are interconnected at different levels and involve crosstalk, which makes their regulatory networks more complex (Song et al., 2017). Low levels of ERS often lead to autophagy; however, under high levels of ERS, autophagy is transiently activated and then accompanied by the activation of apoptosis, along with rapid inhibition of autophagy (Holczer et al., 2015). Additionally, autophagy or proteins involved in the process of autophagy may drive the activation of apoptosis and necrosis by breaking down indispensable components of the cells, which in turn facilitates cell death (Zhan et al., 2020). 3-methyladenine (3-MA), the PI3K inhibitor, was the first identified, and is the most widely used, autophagy inhibitor. Class III PI3K is an activator of autophagy and plays a crucial role in an early step of autophagosome formation in mammalian cells. Studies have confirmed that 3-MA, together with two other PI3K inhibitors, wortmannin and LY294002, suppresses autophagy *via* inhibition of class III PI3K (Yang et al., 2013). Herein, we observed changes in IEC apoptosis and autophagy using CuE combined with the autophagy inhibitor 3-MA. The results showed that CuE promotes Caco-2 cell apoptosis and autophagy; after inhibiting autophagy with 3-MA, the enzymatic hydrolysis of LC3 was inhibited while apoptosis was reduced, but there was no significant effect on CuE-induced ERS. This observation indicates that autophagy is likely to participate in CuE-induced apoptosis of IECs.

**Figure 6:**
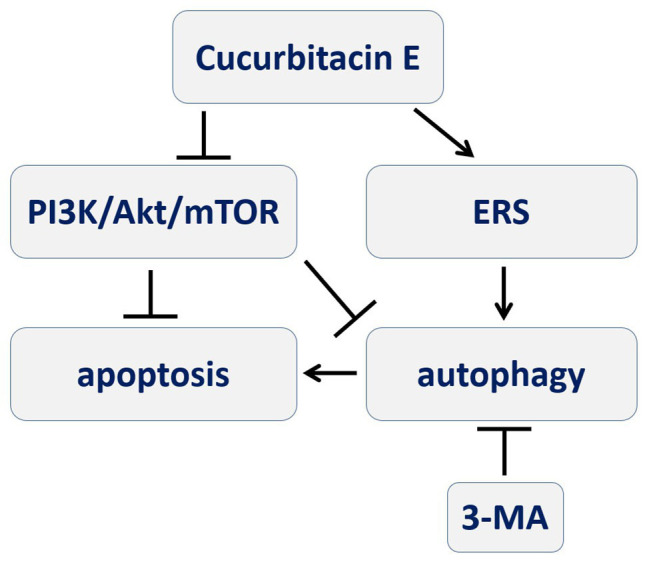
A schematic diagram depicting the process of CuE inducing autophagy-involved apoptosis in IECs that PI3K/AKT/mTOR signal pathway and ERS might be involved, and autophagy participating in pro-apoptotic process.

In conclusion, CuE can induce apoptosis and autophagy in IECs that might be mediated by the PI3K/AKT/mTOR signaling pathway, and autophagy *via* ERS seems to participate in this pro-apoptotic process (Figure 6). It facilitates the study of cofilin in IECs, which is the inhibition target of CuE, in physiological and pathological conditions. Furthermore, CuE could be developed as a potential intervention for intestinal diseases.

## Data Availability Statement

The raw data supporting the conclusions of this article will be made available by the authors, without undue reservation.

## Author Contributions

HSo drafted the manuscript. HSu, QZ, and PW performed parts of the experiments. FW conceived the experiments and revised the manuscript. All authors contributed to the article and approved the submitted version.

### Conflict of Interest

The authors declare that the research was conducted in the absence of any commercial or financial relationships that could be construed as a potential conflict of interest.
